# Abdominoperineal Resection for T4 Low Rectal Cancer After Neoadjuvant Therapy—Are the Outcomes Acceptable?

**DOI:** 10.1007/s13193-024-02028-3

**Published:** 2024-07-19

**Authors:** Devesh S Ballal, Prudvi Raj, M Janesh, Mufaddal Kazi, Ashwin Desouza, Avanish P. Saklani

**Affiliations:** 1 Division of Colon and Rectal Surgery, Advocate Lutheran General Hospital, Park Ridge, IL USA; 2 Division of Colo-Rectal and Peritoneal Surface Oncology, Department of Surgical Oncology, Tata Memorial Hospital, Dr E. Borges Marg, Parel, Mumbai 400012 India; 3 Homi Bhabha National Institute, Mumbai, India

**Keywords:** Response MRI, T4 rectal cancer, APR

## Abstract

**Introduction:**

There is no clear consensus on using the response MRI as opposed to the pretreatment MRI for surgical planning in cT4 low rectal cancer. The objective of this study is to determine the safety of using response MRI in surgical planning for T4 rectal cancer.

**Methods:**

This study is a retrospective review of a prospectively maintained database of abdominoperineal resections conducted at a single tertiary cancer center. Patients undergoing an abdominoperineal resection were divided into 2 groups: group A (clinical T3, mesorectal fascia positive) and group B (clinical T4), and propensity matching was used to account for uneven distribution of baseline characteristics. Primary outcome was the rate of pathological circumferential resection margin positivity. Secondary outcomes were survival outcomes and recurrence patterns.

**Results:**

There were 237 patients in group A and 127 in group B, in the unmatched cohort, with a significantly higher number of females (43.3% vs. 28.7%, *p* = 0.005) and anterior circumferential resection margin positivity (68.5% vs. 49%, *p* < 0.001), with a lower number of patients receiving neoadjuvant chemotherapy in group B (38.6% vs. 49.8%, *p* = 0.041). After propensity matching baseline characters were comparable. There was a higher percentage of extended-total mesorectal excisions in group B (58.5% vs. 40.5%, *p* = 0.004). The rate of pathological circumferential positivity was comparable in both groups (20/168 in group A {11.9%} vs. 13/107 in group B {12.1%}, *p* = 0.951) with no impact of group on circumferential resection margin positivity on univariate (OR 1.023, *p* = 0.951) or multivariate regression (OR 0.993, *p* = 0.987). Both the DFS (median DFS 39 months vs. 54 months, *p* = 0.970) and OS (3-year OS 72% vs. 67%, *p* = 0.798) were comparable between both groups.

**Conclusion:**

For T4 low rectal cancers, post-treatment MRI can be used for surgical planning without any detriment in pathological or long-term oncological outcomes.

**Supplementary Information:**

The online version contains supplementary material available at 10.1007/s13193-024-02028-3.

## Introduction

Neoadjuvant chemoradiation for locally advanced low rectal cancers is now standard of care and has reduced the local recurrence rates significantly to as low as 5–7% [1]; however, there is no clear consensus on the whether to use the pretherapy MRI or response MRI for surgical planning when treating T4 rectal cancer [2–5]. However, the recurrence rates for mesorectal fascia (MRF)-involved rectal cancers is much higher, especially for those patients with other adverse prognostic features with pathological circumferential resection margin (pCRM) positivity reported to 13% for patients without extramural vascular invasion (EMVI) and anterior MRF free but 60% in those patients with both EMVI and anterior CRM involved [6].

To minimize morbidity, many centers, including ours, based surgical planning on the response MRI to avoid unnecessary organ resection [7–9]. While this approach lowers the rate of extended and beyond TME surgeries (e-TME and b-TME, respectively), the oncological control of this approach has not been validated by any large-scale studies. Pathological circumferential resection margin (pCRM) positivity represents the failure of local control, and a higher-than-expected rate would imply that using response MRI as a guide for surgical planning lacks oncological safety. To determine the oncological soundness of utilizing the response MRI for surgical planning, we conducted a review of clinical T4 rectal cancer patients that were sufficiently downstaged using chemoradiation to allow for surgery without additional organ resection, that is excluding patients who underwent a beyond-TME/exenteration. These patients (cT4 patients who underwent abdominoperineal resections {APR} after chemoradiation—group B) were compared with high-risk clinical T3 patients (who had a baseline mesorectal fascia involvement—group A) for whom an APR was performed.

## Materials and Methods

### Study Design

Single-institute retrospective study used data from a prospectively maintained database of patients undergoing APR. Patients undergoing an APR for locally advanced low rectal cancer between June, 2010, and March, 2020, to allow for a 3-year follow-up period, were included in the study. All treatment decisions were taken by a multidisciplinary tumor board and were based on available guidelines at the time. The unit protocol dictates that all cT4 patients that were downstaged sufficiently (≤ ycT3) underwent a non-exenterative surgery. Patients with persistent organ involvement (ycT4) were treated with beyond-TME/extenteration surgeries.

Patients undergoing an APR are known to have worse pathological outcomes compared to those that undergo sphincter-saving surgeries [10] and to eliminate the confounding factors at play when comparing between different surgery types, only low rectal cancers that underwent an APR were included in the study and divided into two groups.

Group A consisted of cT3, MRF-positive patients and group B consisted of cT4 patients. Only patients undergoing standard TME or extended TME (e-TME) surgeries were included while beyond TME surgeries, including exenterations, and patients undergoing wait and watch were excluded.

### End Points

The primary end points were the rate of pCRM positivity with secondary end points being 3-year overall survival (OS), disease-free survival (DFS), and patterns of recurrence. Disease-free survival (DFS) was defined as time from surgery-to-disease recurrence or death due to any cause or last known follow-up whichever was earlier. Overall survival (OS) was defined as time from surgery to death due to any cause or last known follow-up whichever was earlier.

### Propensity Score Matching (PSM)

Logistic regression was used to calculate propensity scores for each patient based on quadrant of CRM involvement, sex, clinical N stage, and surgical approach (minimal invasive surgery {MIS} or open). PSM was then done at a 2:1 ratio and caliper of 0.25.

### Statistical Analysis

SPSS v28.0 by IBM was used for statistical analysis.

Categorical values are presented as percentages and frequencies and compared using the chi-squared test. Continuous variables are presented are medians with interquartile range and compared using the Mann-Whitney U test. Survival curves were plotted using the Kaplan-Meier method and compared using the log-rank test. Follow-up duration was calculated using the reverse Kaplan-Meier method and presented as median duration with interquartile range. Univariate regression was done using binomial logistic regression to identify variables contributing toward a positive CRM, and then a multivariate regression was performed on those factors found to have a significant (*p* < 0.05) impact on univariate analysis.

### Ethics

The study protocol was in accordance with the ethical standards of the institutional research committee. The local Ethics Committee decided to waive the requirement of informed consent because of the retrospective nature of the study and use of anonymized patient data.

This study adheres to the STROBE guidelines.

## Results

**Fig. 1:**
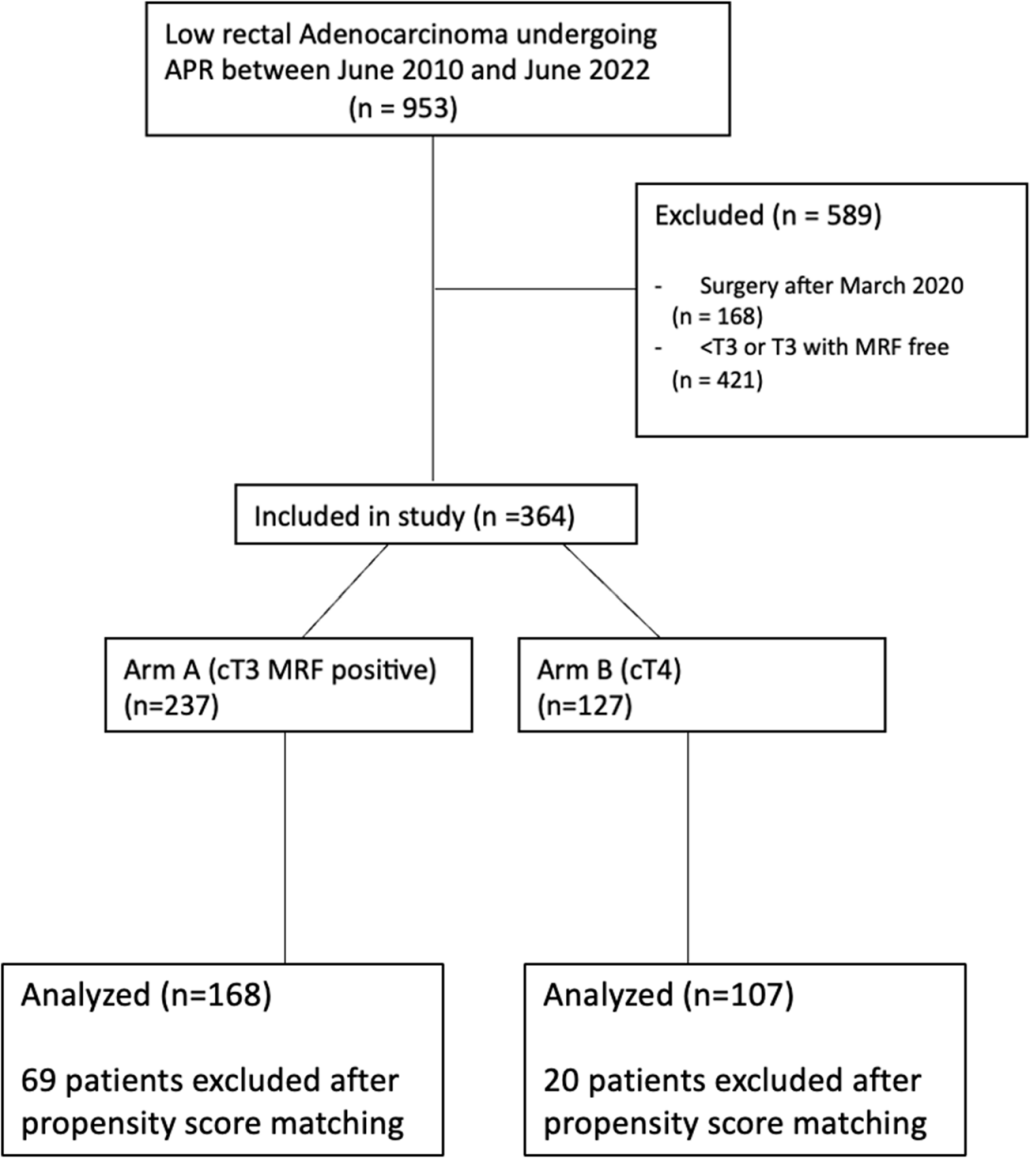
Consort diagram depicted selection of patients for analysis

As depicted in the consort diagram in Fig. 1, a total of 364 patients who underwent an APR between June, 2010, and March, 2020, were included in the analysis. Group A had a lower incidence of female patients (28.7% vs. 43.3%, *p* = 0.005), a lower utilization of neoadjuvant chemotherapy (49.8% vs. 38.6%, *p* = 0.041), and a lower incidence of anterior CRM positivity (49% vs. 68.5%, *p* < 0.001). After propensity matching there was an equal distribution of demographic variables as depicted in Table 1, which shows both the demographic details of both the baseline and matched cohorts.

**Table 1:** Demographic details before and after propensity score matching

	*Baseline*			*After propensity matching*		
	cT3 MRF+, Group A *N* 237	cT4, Group B *N* = 127	*p* value	cT3 MRF+, Group A *N* = 168	cT4, Group B *N* = 107	*p* value
Age (years)	46 (35–56)	48 (35–56)	0.235	47 (35–58)	50 (37–58)	0.470
Gender Male Female	169 (71.3%) 68 (28.7%)	72 (56.7%) 55 (43.3%)	0.005	120 (71.4%) 48 (28.6%)	71 (66.4%) 36 (33.6%)	0.656
BMI (kg/m^2^)	22.5 (20–24.6)	22 (18.9–24.4)	0.378	22.6 (20.2–24.6)	22 (18.9–24.1)	0.245
Quadrant MRF+ Anterior Lateral Posterior Missing data	116 (49%) 83 (35%) 38 (16%)0	87 (68.5%) 20 (15.7%) 17 (13.4%) 3 (2.4%)	< 0.001	103 (61.3%) 43 (25.6%) 22 (13.1%)	70 (65.4%) 20 (18.7%) 17 (15.9%)	0.388
Clinical N stage N0 N1 N2	31 (13%) 105 (44%) 101 (43%)	7 (5%) 63 (50%) 57 (45%)	0.077	12 (7.1%) 70 (41.7%) 86 (51.2%)	7 (6.6%) 44 (41.1%) 56 (52.3%)	0.973
Pre-op M1	11 (6%)	5 (4%)	0.755	2 (3.6%)	5 (4.7%)	0.650
NACT	118 (49.8%)	49 (38.6%)	0.041	81 (48.2%)	45 (42.1%)	0.318
Pre-op RT LCRT SCRT None	187 (84%) 49 (14%) 1 (2.6%)	99 (78%) 28 (22%)	0.734	134 (79.8%) 33 (19.6%) 1 (0.6%)	81 (75.7%) 26 (24.3%)	0.488

Abbreviations: *LCRT*, long course chemoradiotherapy; *SCRT*, short course radiotherapy; *NACT*, neoadjuvant chemotherapy; *MRF*, mesorectal fascia

**Fig. 2:**
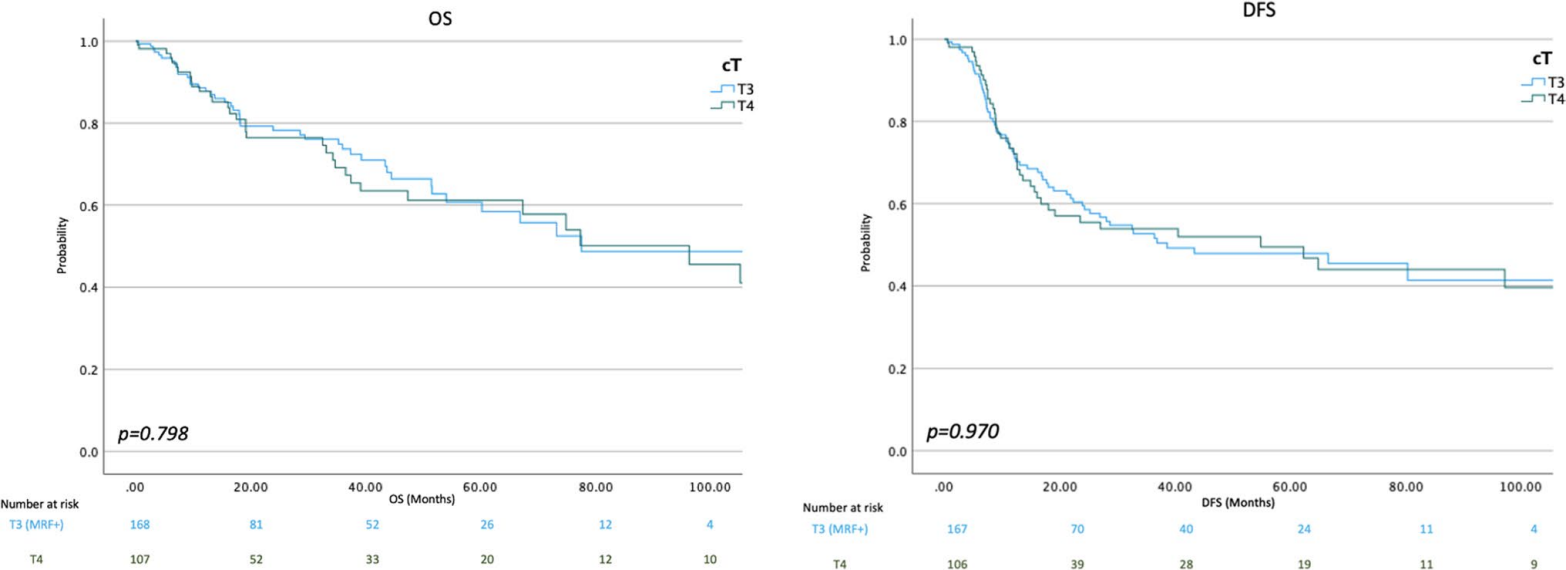
Overall survival (OS) and disease-free survival (DFS)

The intraoperative, post-operative, and oncological outcomes of both groups, before and after matching, are depicted in Table 2. In the matched dataset, group A patients were less likely to undergo e-TME surgeries (40.5% vs. 58.5%, *p* < 0.004) and had a lower pathological T stage (pT4 rate 4.8% vs. 18.7%), which probably represents the higher likelihood of post-treatment downstaging in the cT3 arm. The oncological outcomes and rate of pCRM positivity were comparable between both groups (20/168 in group A {11.9%} vs. 13/107 in group B {12.1%}, *p* = 0.951). The recurrence rates and patterns of recurrence were also comparable between both groups. The OS (3-year OS 72% vs. 67%, *p* = 0.798) and DFS (median DFS 39 months vs. 54 months, *p* = 0.970) were also similar as shown in Fig. 2. The median follow-up for the entire cohort was 35.7 months (interquartile range 9.2–64.5 months).

**Table 2:** Operative details and postoperative and oncological outcomes

	*Baseline*			*After propensity matching*		
Outcome	cT3 MRF+, Group A *N* = 237	cT4, Group B *N* = 127	*p* value	cT3 MRF+, Group A *N* = 168	cT4, Group B *N* = 107	*p* value
e-TME	89 (38%)	71 (56%)	< 0.001	68 (40.5%)	62 (58.5%)	0.004
Hospital stay (days)	8 (6–10)	8 (6–10)	0.97	7 (5–9)	7.5 (6–10)	0.1
CD ≥ 3a	36 (15%)	23 (18%)	0.886	32 (19%)	20 (18.9%)	0.672
Blood loss (mL)	500 (300–800)	600 (400–1000)	0.011	500 (300–913)	600 (400–1000)	0.156
Approach Laparoscopy Robotic Open Conversion to open	141 (59%) 19 (8%) 75 (32%) 2 (1%)	51 (40%) 8 (6%) 67 (53%) 1 (1%)	< 0.001	86 (51.2%) 11 (6.5%) 70 (41.7%) 1 (0.6%)	47 (44%) 7 (6.5%) 52 (48.6%) 1 (0.9%)	0.429
pT stage pT1 pT2 pT3 pT4 pT0	5 (2%) 40 (17%) 130 (55%) 10 (4%) 52 (22%)	5 (4%) 20 (16%) 57 (45%) 24 (18%) 21 (17%)	0.003	1 (0.6%) 28 (16.7%) 98 (58.3%) 8 (4.8%) 33 (19.6%)	3 (2.8%) 17 (15.9%) 49 (45.8%) 20 (18.7%) 18 (17.7%)	0.011
pCRM Negative Positive	215 (91%) 22 (9%)	111 (87%) 16 (13%)	0.324	148 (88.1%) 20 (11.9%)	94 (87.9%) 13 (12.1%)	0.951
Recurrence^#^ None Local alone Systemic Local + systemic Peritoneal	163 (69%) 18 (8%) 40 (17%) 3 (1%) 14 (6%)	80 (63%) 7 (5.5%) 25 (20%) 3 (3%) 11 (9%)	0.264	110 (65.5%) 14 (8.3%) 31 (18.5%) 3 (1.8%) 12 (7.1%)	67 (62.6%) 5 (4.7%) 23 (21.5%) 1 (0.9%) 10 (9.3%)	0.629
Deaths	92 (30%)	36 (28%)	0.206	42 (25%)	32 (29.9%)	0.371
Follow-up (months)	33 (7.6–57)	41 (13.3–76.9)	0.002	35.7 (29–38.5)	35.4 (12.5–50.7)	0.025

^#^ Some patients developed multiple sites of recurrence—numbers do not add to 100%

Abbreviations: *CD*, Clavien-Dindo complication; *e-TME*, extended TME; *pCRM*, pathological circumferential resection margin

On logistic regression, factors that significantly impacted pCRM positivity were male gender, open approach, not receiving NACT, and clinical nodal stage as shown in Table 3. On multivariate analysis only female gender (OR 0.217, *p* = 0.017) and open approach (OR 2.711, *p* = 0.022) were found to impact CRM positivity. The group (cT3 MRF+ or cT4) did not impact the CRM positivity either on univariate or multivariate analysis. Regression analysis on unmatched data also showed similar findings, as seen in Supplementary Table S1.

**Table 3:** Logistic regression for factors resulting in pCRM positivity on propensity matched data

Factor	Univariate logistic regression			Multivariate regression		
	OR	95% CI	*p* value	OR	95% CI	*p value*
Female gender	0.199	0.059–0.671	0.009	0.217	0.062–0.759	0.017
BMI	1.096	0.979–1.226	0.111	NA		
Age	0.985	0.96–1.01	0.239	NA		
NACT not received	2.283	1.075–4.850	0.032	1.93	0.869–4.307	0.106
Approach Lap/robotic Open	1 3.02	Reference 1.371–6.652	0.006	1 2.711	Ref 1.152–6.381	0.022
Group A (cT3 MRF+) B (cT4)	1 1.023	Reference 0.486–2.155	0.951	1 0.993	Ref 0.442–2.232	0.987
cN stage N0 N1 N2	1 4.304 1.364	Ref 0.545–34 0.165–11.29	0.011	1 2.253 1.168	Ref 0.267–19.01 0.136–10.025	0.29
RT type LCRT SCRT	1 2.1	Reference 0.956–4.688	0.181	NA		
CEA	1.001	0.997–1.005	0.595			
CRM involvement Anterior Lateral Posterior	1 0.858 0.784	Reference 0.347–2.119 0.254–2.421	0.886			

Abbreviations: *LCRT*, long-course chemoradiotherapy; *SCRT*, short-course radiotherapy; *NACT*, neoadjuvant chemotherapy; *MRF*, mesorectal fascia

## Discussion

Our results showed that the outcomes of cT4 rectal cancers undergoing APR after downstaging with chemoradiation are comparable to those of cT3, MRF-positive tumors in terms of pathological CRM positivity, local recurrence, DFS, and OS.

The importance of response MRI-based planning was highlighted in the MERCURY II study, which showed that no patient in whom a response MRI showed a safe resection plane (ymrLRP) had a pathologically involved CRM [6]. Our results show acceptable rates of pCRM positivity in a subset of high-risk low rectal cancer with a pCRM positivity rate of 11.9% in group A and 12.1% in group B, despite the slightly higher incidence of anterior MRF involvement in group B which is a known adverse prognostic factor [6]. The rates of local recurrence are also low – 10.1% in group A and 5.6% in group B, with no significant difference between the two arms.

Despite the lower rate of e-TME (40.5% vs. 58.5%, *p* < 0.001) and blood loss (median blood loss 500 vs. 600 mL, *p* = 0.156) in the T3 MRF+ arm, there was comparable post-op morbidity (CD grade ≥ 3a 19% vs. 18.9%, *p* = 0.672) and the quality of life and complication rate are significantly better than in patients undergoing beyond TME resections/exenterations [11, 12]. Previous studies by the authors have demonstrated the safety and feasibility of such e-TME resections [13, 14], the technique which has also been previously published [15], which are feasible by minimally invasive approaches and lead to comparable oncological outcomes when compared to exenterations [16].

The regression analysis performed showed that cT stage did not impact pCRM positivity or oncological outcomes. Only female sex and open approach had an impact on CRM positivity on multivariate analysis. The lower incidence of pCRM positivity in females can be explained by the fact that an e-TME with posterior vaginal wall excision can be performed easily and without much added morbidity in cases with a doubtful anterior CRM in females, but the same situation does not exist in males when additional margins would usually require resection of either the prostate or bladder. The association of open surgery with a positive pCRM can be explained by an inherent selection bias as a MIS approach is usually the preferred approach due to the better visualization provided.

Given the difficulty in performing surgical trials and impracticality of a randomized trial comparing exenterations to e-TME resections, evidence to support the use of response MRI for surgical planning will only be retrospective in nature. Given the comparable OS and DFS between both arms in our study and the excellent local control rates for this group of high-risk rectal cancer, it does not seem logical to base surgical planning on pretreatment imaging which will lead to a much higher rate of exenterations. Response to chemoradiation is a known good prognostic factor [17] and treatment of these patients expected to have good long-term outcomes should factor in long-term quality of life while formulating the surgical plan [2].

There are a few limitations of this retrospective study. Being a retrospective review, a significant selection bias exists which confounds the interpretation of the results, and while we have attempted to balance the baseline differences between groups, including rates of consolidation chemotherapy, using propensity matching, this may still have skewed the results as delivery of total neoadjuvant therapy has been shown to improve the DFS in locally advanced rectal cancer [18, 19]. While a synoptic radiologic and pathologic system is now employed for all cases, EMVI and other significant prognostic information are missing in the initial period of data collection, preventing a more meaningful comparison and propensity matching between cohorts.

## Conclusion

For T4 low rectal cancers, post-treatment MRI can be used for surgical planning without any detriment in either pathological or long-term oncological outcomes.

## Supplementary Information

Below is the link to the electronic supplementary material.

Supplementary file1 (DOCX 92 KB)

## Data Availability

Anonymized patient data used in this study is available on request from the authors.
