# Supplementary material for: Abdominoperineal Resection for T4 Low Rectal Cancer After Neoadjuvant Therapy—Are the Outcomes Acceptable?
Source: Indian J Surg Oncol. 2024 Jul 19;15(4):612–8. doi: 10.1007/s13193-024-02028-3 (PMC11564435; doi:10.1007/s13193-024-02028-3)
Supplement: Supplementary file 1 — Supplementary file1 (DOCX 92 KB) [file 13193_2024_2028_MOESM1_ESM.docx]

Supplementary Table S1 - Univariate and Multivariate Regression for factors influencing CRM positivity on baseline data.

| Factor | Univariate Logistic Regression | | |  | Multivariate regression | | |
| --- | --- | --- | --- | --- | --- | --- | --- |
|  | OR | 95% CI | p value |  | OR | 95% CI | *p value* |
| Female Gender | 0.31 | 0.096 - 0.995 | **0.049** |  | 0.282 | 0.1 – 0.796 | **0.017** |
| BMI | 1.17 | 1.034 - 1.326 | **0.013** |  | 1.088 | 0.98 – 1.208 | 0.113 |
| Age | 0.97 | 0.935 – 1.0 | 0.053 |  | 0.985 | 0.958 – 1.014 | 0.306 |
| Approach   - Lap/robotic - Open | 1  2.783 | Reference  1.380 – 5.613 | **0.004** |  | 1  2.933 | Ref  1.283 – 6.706 | **0.011** |
| Group   - A (cT3 MRF+) - B (cT4) | 1  1.55 | Reference  0.547-4.371 | 0.411 |  | 1  0.855 | Ref  0.355 – 2.06 | 0.727 |
| cN stage   - N0 - N1 - N2 | 1  6.78  2.8 | Ref  0.89 - 51.57  0.35 – 22.13 | **0.017** |  | 1  3.566  2.32 | Ref  0.44 – 28.72  0.28 – 19.24 | 0.35 |
| RT type   - NACTRT - SCRT | 1  1.959 | Reference  0.632-6.076 | 0.244 |  |  | | |
| NACT received | 0.61 | 0.2 – 1.87 | 0.387 |  |  |  |  |
| CEA | 1.002 | 0.998 – 1.006 | 0.381 |  |  |  |  |
| CRM involvement   - Anterior - Lateral - Posterior | 1  0.496  0.681 | Reference  0.208 – 1.186  0.249 – 1.864 | 0.261 |  |  |  |  |
